# Response to important considerations when assessing the effect of essential fatty acids on cognitive performance

**DOI:** 10.1186/s12937-020-00620-1

**Published:** 2020-09-11

**Authors:** Xue Dong, Dongfeng Zhang

**Affiliations:** Department of Epidemiology and Health Statistics, The School of Public Health of Qingdao University, No. 308 Ningxia Road, Qingdao, 266021 China

**Keywords:** ω-3 fatty acids, ω-6 fatty acids, Nutrition, Cognitive performance, NHANES

## Abstract

In this letter, we respond to the comments raised by Visaria et al. in their letter to the editor about the publication “Association of dietary ω-3 and ω-6 fatty acids intake with cognitive performance in older adults: National Health and Nutrition Examination Survey (NHANES) 2011–2014”. We have further adjusted for some key covariates as the authors mentioned in the letter and performed sensitivity analysis by excluding vegetarians considering the bioavailability of fatty acids from different sources. In conclusion, the results were basically consistent with our previous results, which showed that the results were stable and reliable. We hope that our study could be helpful in further studies delineating the various intricacies of fatty acid nutrition and metabolism and control for covariates.

To the Editor:

We would like to thank you for the opportunity to respond to the comments raised by Visaria et al. in their letter to the editor about the publication “Association of dietary ω-3 and ω-6 fatty acids intake with cognitive performance in older adults: National Health and Nutrition Examination Survey (NHANES) 2011–2014”. We would also like to thank Visaria et al. for their interest in our paper and for taking the time to express their views. We will answer the questions in detail about covariates and methodological considerations raised by the authors.

Visaria and colleagues’ first comment pertains to the control of covariates. They contend that while we have appropriately controlled for demographic, socioeconomic, physical activity, and cardiometabolic comorbidity factors, there are several other covariates that need to be accounted for, such as vitamin, mineral and other dietary consumption, sociobehavioral risk factors and physical & mental comorbidities. According to their suggestion and our sample size (2496 participants), we further adjusted for vitamin D [1], niacin [2], vitamin B6 [3], vitamin B12, folic acid, zinc [4], iron, copper, selenium, protein [5], total saturated fatty acids [6], and depression [7] in Model 2. Table 1 presents the characteristics of the study population across cognitive status. There were no significant differences between people with low cognitive performance and normal cognitive performance in the distribution of pre-diabetes, gastrointestinal disorders, and smoking among three tests. So, we did not put them into the overall analysis. Table 2 shows the associations of ω-3 fatty acids, ω-6 fatty acids, and ω-6: ω-3 ratio with three tests. In the full-adjusted model, the odds ratios (ORs) with 95% confidence interval (CI) of the Consortium to Establish a Registry for Alzheimer’s disease (CERAD) test score, Animal Fluency test score and the Digit Symbol Substitution test (DSST) score were 0.59(0.38–0.92), 0.69(0.46–1.06) and 0.57(0.39–0.83) for the highest versus lowest tertile of ω-3 fatty acids, respectively; the ORs with 95% CI of CERAD test score, Animal Fluency test score and DSST score were 0.50(0.32–0.79), 0.64(0.42–0.98) and 0.55(0.37–0.81) for the highest versus lowest tertile of ω-6 fatty acids, respectively. The association between ω-6: ω-3 ratio and cognitive performance was not statistically significant in three tests. Although the association between ω-3 fatty acids and Animal Fluency test did not reach statistical significance, the OR of Animal Fluency test was 0.69. Overall, the above results were basically consistent with our previous results [8], which showed that the results were stable and reliable.

**Table 1:** Characteristics of the study population, NHANES 2011–2014 (*N* = 2496)

	CERAD test			Animal Fluency test			Digit Symbol test		
	Normal Cognitive Performance	Low Cognitive Performance	*P* Value	Normal Cognitive Performance	Low Cognitive Performance	*P* Value	Normal Cognitive Performance	Low Cognitive Performance	*P* Value
Number of subjects	1851 (74.2)	645 (25.8)		1778 (71.2)	718 (28.8)		1850 (74.1)	646 (25.9)	
Vitamin D (mcg)^b^	3.65 (4.2)	3.64 (3.7)	0.875	3.8 (4.1)	3.35 (3.9)	< 0.01	3.7 (4.2)	3.5 (3.6)	0.042
Niacin (mg)^b^	21.02 (11.8)	19.48 (11.7)	< 0.01	21.33 (11.9)	19.1 (10.6)	< 0.01	21.29 (11.7)	18.71 (11.5)	< 0.01
Vitamin B6 (mg)^b^	1.79 (1.04)	1.64 (1.02)	< 0.01	1.8 (1.06)	1.63 (1.01)	< 0.01	1.81 (1.03)	1.56 (1.04)	< 0.01
Vitamin B12 (mcg)^b^	3.82 (3.3)	3.40 (3.2)	< 0.01	3.88 (3.34)	3.34 (3.2)	< 0.01	3.9 (3.3)	3.15 (3.2)	< 0.01
Folic acid (mcg)^b^	132 (141)	119 (125)	0.012	132 (143)	120 (118)	< 0.01	133 (143)	113 (115)	< 0.01
Zinc (mg)^b^	9.4 (5.3)	8.7 (5.2)	< 0.01	9.62 (5.4)	8.23 (5.1)	< 0.01	9.56 (5.1)	8.05 (4.6)	< 0.01
Iron (mg)^b^	12.94 (7.4)	12.01 (7.7)	< 0.01	13.06 (7.5)	11.91 (7.4)	< 0.01	13 (7.5)	11.7 (7.6)	< 0.01
Copper (mg)^b^	1.08 (0.6)	1.01 (0.6)	< 0.01	1.11 (0.61)	0.95 (0.5)	< 0.01	1.12 (0.59)	0.93 (0.54)	< 0.01
Selenium (mcg)^b^	96.75 (53)	90.85 (52)	< 0.01	99.1 (54)	87.07 (52)	< 0.01	98.62 (51)	85.52 (55)	< 0.01
Protein (gm)^b^	70.06 (34.2)	65.52 (35.9)	< 0.01	71.11 (35.1)	63.73 (33.4)	< 0.01	70.98 (34.0)	61.01 (37.4)	< 0.01
Total saturated fatty acids (gm)^b^	20.65 (14.4)	17.85 (13.7)	< 0.01	21.03 (14)	17.39 (13.4)	< 0.01	21.13 (14)	16.8 (13.8)	< 0.01
Depression (%)^a^	145 (7.9)	71 (11.2)	0.010	120 (6.8)	96 (13.6)	< 0.01	120 (6.5)	96 (15.1)	< 0.01
Pre-diabetes (%)^a^	128 (9.4)	28 (6.4)	0.055	109 (8.2)	47 (10.0)	0.229	130 (9.3)	26 (6.5)	0.074
Gastrointestinal disorders (%)^a^	116 (6.3)	38 (5.9)	0.756	107 (6.0)	47 (6.6)	0.618	111 (6.0)	43 (6.7)	0.537
Smoking (%)^a^	928 (50.2)	333 (51.6)	0.521	908 (51.1)	353 (49.2)	0.382	926 (50.1)	335 (51.9)	0.437

Data are number of subjects (percentage) or medians (inter quartile ranges)

^a^ Chi-square test was used to compare the percentage between participants with and without low cognitive performance

^b^ Mann-Whitney *U* test was used to compare the mean values between participants with and without low cognitive performance

**Table 2:** Weighted odds ratios (95% confidence intervals) for score on CERAD test, Animal Fluency test, and DSST across tertiles of dietary ω-3 and ω-6 fatty acids intake and ω-6: ω-3 ratio, NHANES 2011–2014 (*N* = 2496)

	CERAD test^a^	Animal Fluency test^a^	DSST^a^
ω-3 (mg/kcal/day)			
<0.727	1.00 (Ref.)	1.00 (Ref.)	1.00 (Ref.)
0.727 to <1.04	0.67 (0.46–0.96) ^*^	0.78 (0.55–1.10)	0.97 (0.57–1.65)
≥ 1.04	0.59 (0.38–0.92) ^*^	0.69 (0.46–1.06)	0.57 (0.39–0.83) ^**^
ω-6 (mg/kcal/day)			
<6.538	1.00 (Ref.)	1.00 (Ref.)	1.00 (Ref.)
6.538 to <8.848	0.59 (0.40–0.89) ^*^	0.69 (0.47–1.03)	0.83 (0.54–1.27)
≥ 8.848	0.50 (0.32–0.79) ^**^	0.64 (0.42–0.98) ^*^	0.55 (0.37–0.81) ^**^
ω-6: ω-3 ratio			
<7.684	1.00 (Ref.)	1.00 (Ref.)	1.00 (Ref.)
7.684 to <9.462	0.92 (0.64–1.31)	1.21 (0.83–1.76)	1.10 (0.69–1.75)
≥ 9.462	0.98 (0.61–1.57)	1.19 (0.73–1.93)	0.90 (0.54–1.51)

Model adjusted for age, gender, race, educational level, marital status, income, BMI, recreational activity, work activity, vitamin D, niacin, vitamin B6, vitamin B12, folic acid, zinc, iron, copper, selenium, protein, total saturated fatty acids, drinking status, hypertension, diabetes, stroke, and depression

^*^p < 0.05; ^**^p < 0.01

^a^Calculated using binary logistic regression

The second comment outlined by Visaria et al. in their letter to the editor is the type and source of fatty acid (plant-based vs. animal-based), in context of an individual’s overall dietary patterns. As the authors mentioned, the source of the fatty acid can impact how it is metabolized in the body and its resulting bioavailability. We agree with the authors’ point and we divided participants into two groups (2146 non-vegetarians and 350 vegetarians). However, the question “Do you consider yourself to be a vegetarian?” was only asked in NHANES 2007–2010 [9], so we used two 24-h dietary recall to assess the types of foods the participants ate. Furthermore, considering that vegetarians made up only 14% of the participants, we performed sensitivity analysis by excluding vegetarians in Table 3. In the fully adjusted model, the negative associations of CERAD test and DSST with ω-3 fatty acids and ω-6 fatty acids were still significant. The association of Animal Fluency test with ω-3 fatty acids and ω-6 fatty acids were not statistically significant.

**Table 3:** Weighted odds ratios (95% confidence intervals) for score on CERAD test, Animal Fluency test, and DSST across tertiles of dietary ω-3 and ω-6 fatty acids intake and ω-6: ω-3 ratio excluding vegetarians^b^, NHANES 2011–2014 (*N* = 2146)

	CERAD test^a^	Animal Fluency test^a^	DSST^a^
ω-3 (mg/kcal/day)			
<0.727	1.00 (Ref.)	1.00 (Ref.)	1.00 (Ref.)
0.727 to <1.04	0.61 (0.38–0.95) ^*^	0.77 (0.53–1.13)	0.91 (0.52–1.58)
≥ 1.04	0.55 (0.35–0.88) ^*^	0.69 (0.44–1.06)	0.60 (0.43–0.84) ^**^
ω-6 (mg/kcal/day)			
<6.538	1.00 (Ref.)	1.00 (Ref.)	1.00 (Ref.)
6.538 to <8.848	0.55 (0.35–0.87) ^*^	0.72 (0.50–1.04)	0.93 (0.58–1.50)
≥ 8.848	0.48 (0.30–0.76) ^**^	0.68 (0.44–1.06)	0.56 (0.38–0.79) ^**^
ω-6: ω-3 ratio			
<7.684	1.00 (Ref.)	1.00 (Ref.)	1.00 (Ref.)
7.684 to <9.462	1.00 (0.67–1.48)	1.21 (0.86–1.69)	1.12 (0.73–1.73)
≥ 9.462	1.03 (0.61–1.76)	1.37 (0.79–2.36)	0.87 (0.53–1.45)

Model adjusted for age, gender, race, educational level, marital status, income, BMI, recreational activity, work activity, vitamin D, niacin, vitamin B6, vitamin B12, folic acid, zinc, iron, copper, selenium, protein, total saturated fatty acids, drinking status, hypertension, diabetes, stroke, and depression

^*^p < 0.05; ^**^p < 0.01

^a^Calculated using binary logistic regression

^b^Vegetarians are defined based on two 24-h dietary recall

Regarding the comment from Visaria et al. that variability of 24-h dietary recall from first interview to second interview need to be considered to get an idea of an individual’s dietary consistency, we performed correlation test between the two recalls. We found that the correlation between the two recalls was significant (correlation coefficient: 0.260 for ω-3 fatty acids, 0.295 for ω-6 fatty acids), so we used a combination of the first-day and second-day mean values to make use of all available dietary data. Furthermore, some studies have shown that two 24-h recalls might be sufficient to assess the daily dietary intake [10]. In addition, this self-recall might be affected by one’s cognitive function and lead to potential bias as the authors noted, we agree with the authors and will discuss the limitation for the method in further studies.

## Data Availability

The datasets supporting the conclusions of this article are available in publicly repository as described below. The authors do not own the data. National Health and Nutrition Examination Survey data are available from the National Center for Health Statistics (http://www.cdc.gov/nchs/nhanes/ nhanes_questionnaires.htm).

## Abbreviations

NHANES: The National Health and Nutrition Examination Surveys
CERAD: The Consortium to Establish a Registry for Alzheimer’s disease
DSST: The Digit Symbol Substitution Test
OR: Odds ratio
CI: Confidence interval
